# From Stone to Unknown: Incidental Discovery of Oral Synovial Sarcoma in a Patient With a Renal Staghorn Stone

**DOI:** 10.7759/cureus.96667

**Published:** 2025-11-12

**Authors:** Anwar A AlSuwailem, Rihab Molah, Hatem Khoja, M. Anas Dababo

**Affiliations:** 1 Pathology and Laboratory Medicine, King Faisal Specialist Hospital and Research Centre, Riyadh, SAU

**Keywords:** fish, incidental, oral, radiation, synovial sarcoma

## Abstract

Synovial sarcoma (SS) of the oral cavity is an exceedingly rare malignancy, particularly when arising in the floor of the mouth. We report a case of a 66-year-old male patient who was incidentally found to have a painless mass in the floor of the mouth during a perioperative assessment for nephrolithiasis. Histological examination revealed a biphasic tumor composed of spindle-shaped cells and epithelial elements with neuroendocrine-like immunophenotypic features. Immunohistochemistry confirmed the diagnosis with strong positivity for TLE-1, EMA, and vimentin, and focal positivity for synaptophysin and CD56. Fluorescence in situ hybridization (FISH) confirmed SS18 gene rearrangement.

This case contributes to the limited literature on oral SS, particularly involving the floor of the mouth, and highlights an unusual clinical presentation in an elderly patient with neuroendocrine differentiation. To our knowledge, this is the first reported case of oral SS in Saudi Arabia or the surrounding region. The report underscores the importance of multidisciplinary collaboration in identifying clinically silent yet aggressive tumors.

## Introduction

Synovial sarcoma (SS) is a malignant soft tissue neoplasm of uncertain differentiation. It is often referred to as a spindle cell neoplasm with variable epithelial components [1]. The biphasic pattern, characterized by the coexistence of spindle and epithelial cells, is a hallmark feature, though both populations are histologically related [2]; hence, the suggestion of “spindle cell carcinoma or carcinosarcoma of soft tissue” [3]. SS predominantly affects young adults, with only 1.6% of cases occurring in patients over 50 years of age. There is a slight male predominance, with a male-to-female ratio of approximately 7:5 [4]. Most cases arise in the soft tissues of the extremities, with only 3% occurring in the head and neck region, making oral SS exceedingly rare [3]. When located in the head and neck, SS may present with compression symptoms or changes in voice rather than the typical palpable mass seen in the extremities [5].

## Case presentation

A 66-year-old male patient with a history of hypertension, diabetes mellitus, and depression was found to have a left renal staghorn calculus. He had been under follow-up at a local hospital for two months, with laboratory investigations revealing normal renal function. A dimercaptosuccinic acid scan demonstrated multiple cortical parenchymal defects in the left kidney.

**Figure 1:**
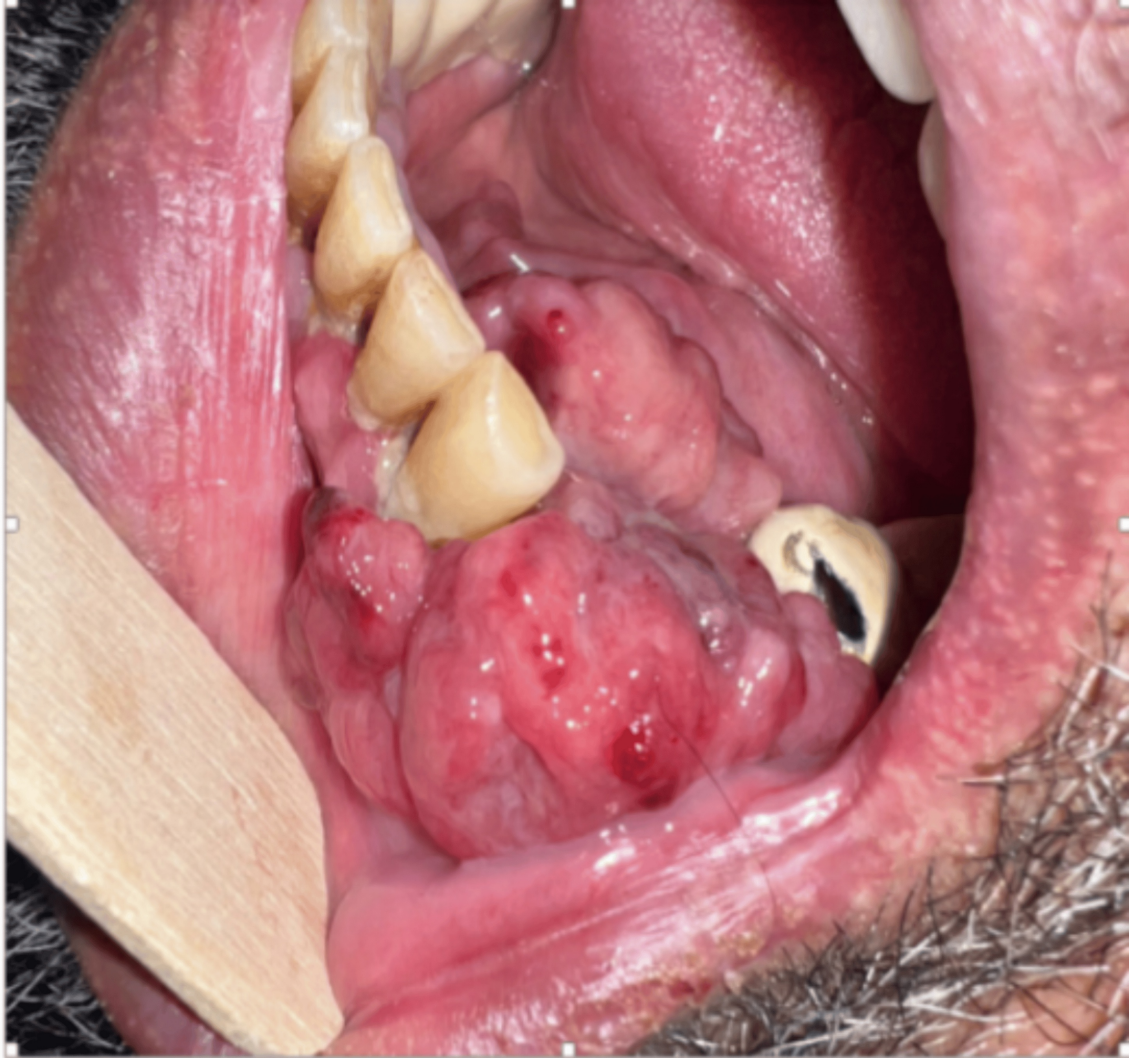
A clinical photograph of a left lower alveolar mass extending to the floor of the mouth, approximately 5 cm in size, fungating, malodorous, and painless.

**Figure 2:**
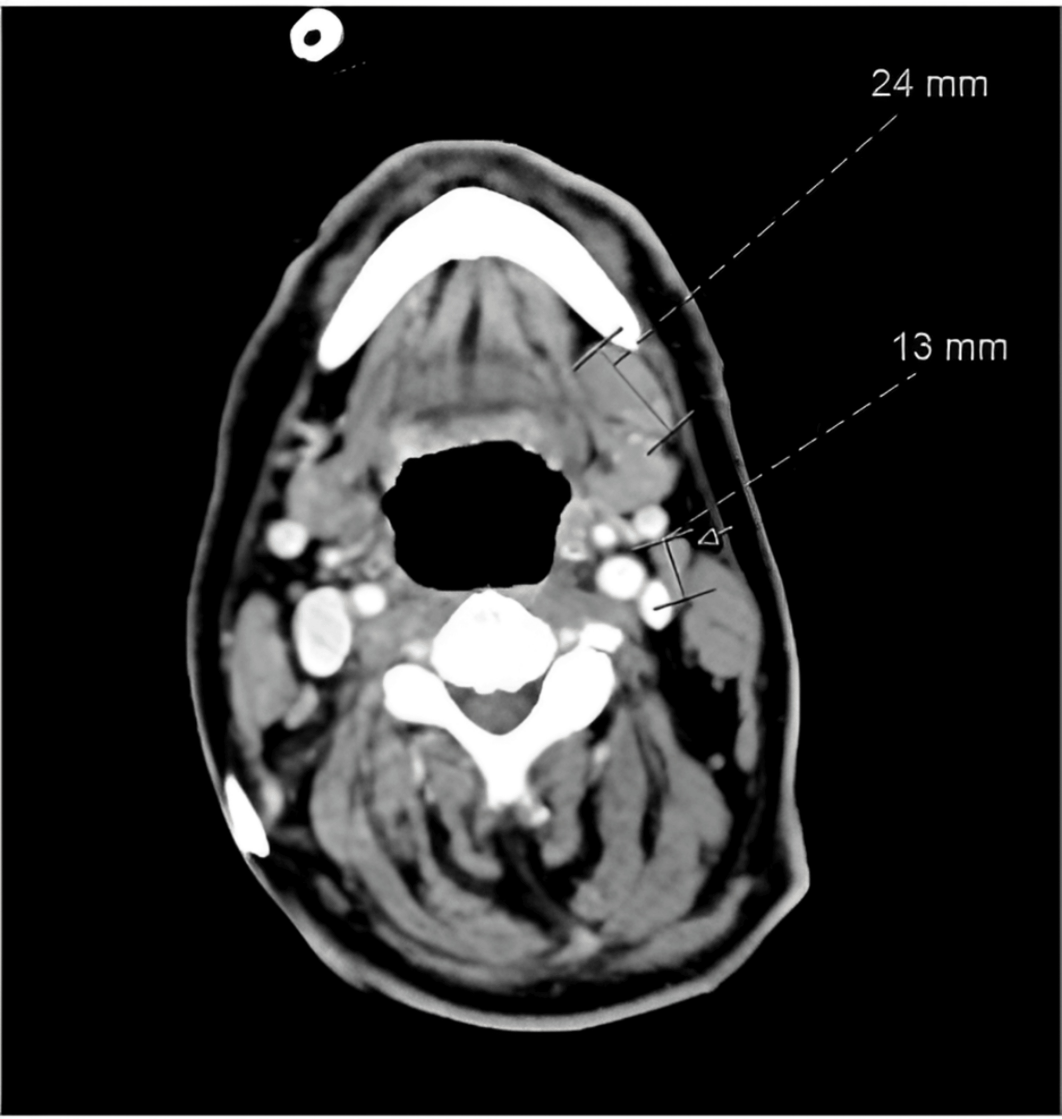
The post-biopsy CT showed an alveolar mucosal-associated soft tissue mass with some enlarged neck lymph nodes.

During preoperative assessment, an incidental 5 cm mass was noted by the anesthesiology team in the left lower oral alveolus, extending to the floor of the mouth. The lesion was fungating, malodorous, and painless (Figure 1). Following completion of his nephrolithotomy, a consultation with the ENT team was requested, and they proceeded with a biopsy and a whole-body computed tomography scan (Figure 2). 

After the histopathology biopsy result, the patient subsequently underwent palliative radiotherapy, as he was deemed to be unfit for surgery or a candidate for chemotherapy due to his multiple comorbidities, as decided by the oncology team.

**Figure 3:**
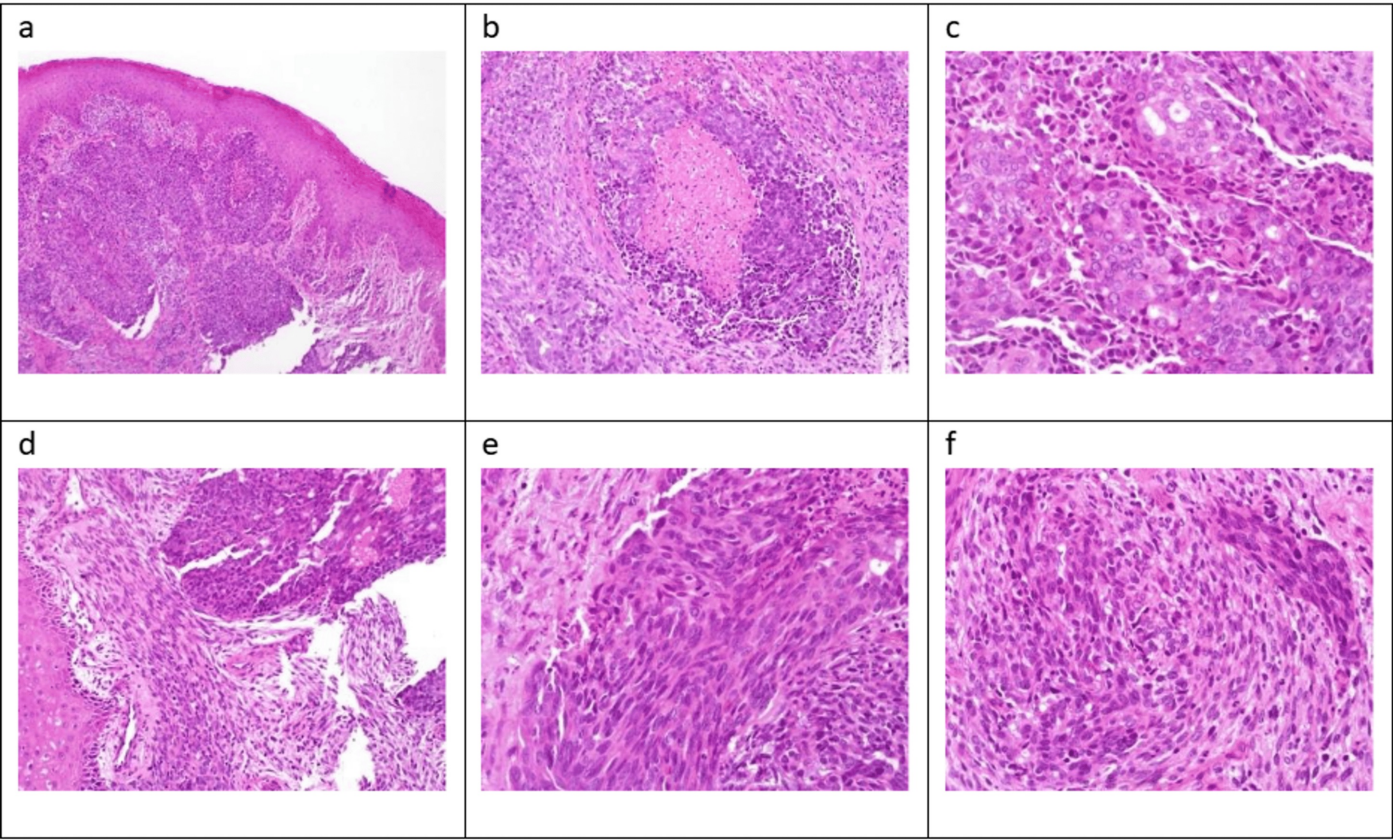
Histologic view (a) Squamous surface epithelium with underlying infiltrative lesion composed of nests of squamous cells as well as other epithelial nests (H&E stain ×10). (b) Some of the nests show central necrosis surrounded by atypical cells with eosinophilic cytoplasm and vesicular chromatin (H&E stain ×20). (c) In some areas, these nests show gland-like spaces with central lumina (H&E stain ×40). (d) In some areas, adjacent to these nests, a spindle cell proliferation (H&E stain ×20). (e) These spindle cell areas are also highly atypical and mitotically active (H&E stain ×40). (f) Epithelial and spindle cells merge together (H&E stain ×40).

Histopathological examination of the oral lesion biopsy revealed focal surface ulceration with infiltrative nests of highly pleomorphic and mitotically active squamous and epithelial-like cells, some with central necrosis. Spindle cell proliferation and areas of neuroendocrine differentiation are noted (Figure 3). Our morphological differential diagnoses were poorly differentiated squamous cell carcinoma, poorly differentiated adenocarcinoma, among adenosquamous carcinoma with neuroendocrine differentiation, adenosquamous carcinoma with sarcomatoid components, human papillomavirus (HPV)-associated squamous cell carcinoma, and malignant peripheral nerve sheath tumor (MPNST) with divergent differentiation.

**Figure 4:**
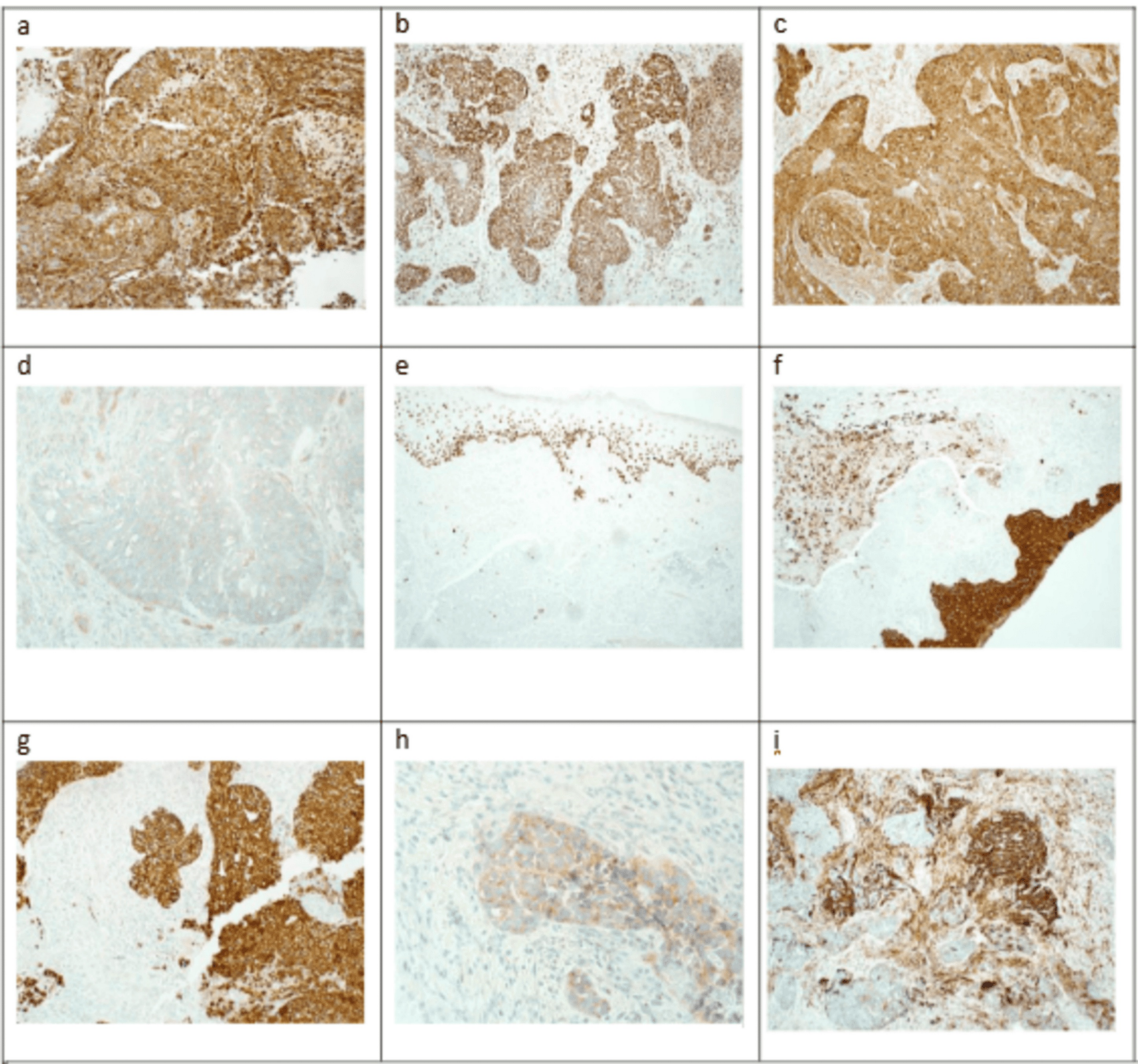
Immunohistochemistry view (a) The tumor cells are diffusely positive for vimentin. (b) Strong nuclear positivity for TLE-1 (c) and (d) weak positivity for BCL2 and CD99, respectively (e-g) and (e-h), and (i) focally positive for P63, CK5/6, CK7, synaptophysin, and CD56 (IHC a, b, c, e, f, and i ×10, d and g ×20, h ×40).

**Figure 5:**
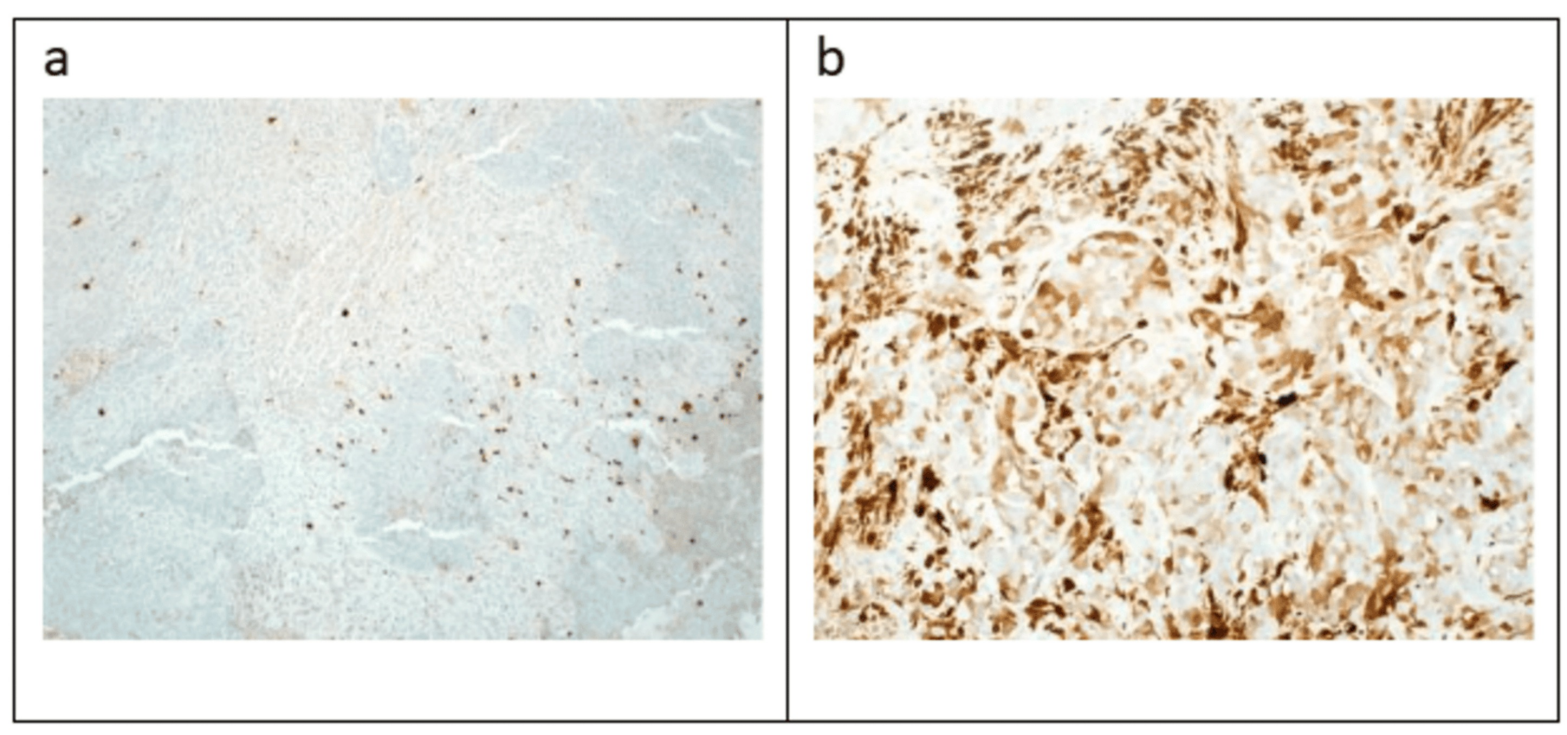
Immunohistochemistry (IHC) view (a) The tumor cells are negative for S100 (IHC x10) and (b) P16, which showed less than 70% block staining (IHC x20).

Immunohistochemical analysis showed diffuse positivity for vimentin. The tumor also showed focal positivity for p53, p63, CK5/6, CK7, synaptophysin, and CD56, and mucicarmine, which supports the inclusion of adenosquamous carcinoma with neuroendocrine and sarcomatoid components. While they turned negative for S100 and p1,6, which excludes MPNST and HPV-associated squamous cell carcinoma (Figures 4, 5).

**Figure 6:**
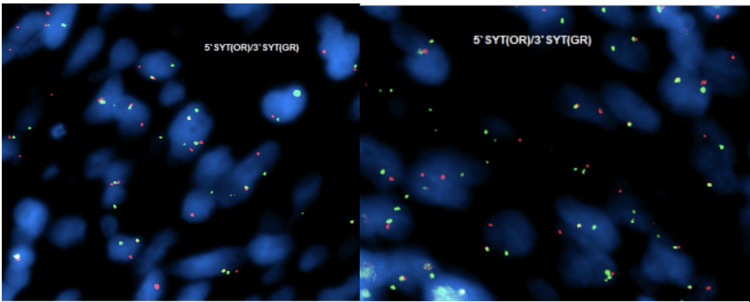
SS18 gene (18q11.2) rearrangement in 93% of tumore nuclei

TLE-1 was done in the first round of immunohistochemical panels and showed strong nuclear expression. In addition to weak positivity for BCL2 and CD99. Therefore, fluorescence in situ hybridization (FISH) analysis was requested, and it was done using the SS18(SYT)(8q11.2) break-apart probe (Vysis, Abbott Molecular, Inc., Des Plaines, IL). One hundred nuclei were scored manually from a paraffin-embedded tissue sample, confirming the SS18 gene (18q11.2) rearrangement in 93% of nuclei, establishing the diagnosis of SS (Figure 6).

Despite starting palliative radiotherapy, the patient did not tolerate treatment and passed away from cardiopulmonary arrest nine months after diagnosis under palliative care.

## Discussion

SS is a rare mesenchymal malignancy of unknown histological origin [6], typically affecting the extremities of young adults. However, head and neck involvement is uncommon, with only 3% of cases occurring in this region, and even fewer reported in the oral cavity. The floor of the mouth is an especially rare location, accounting for less than 5% of the more than 250 reported cases of head and neck SS in the literature [1,7]. 

Histologically, the classic morphology of SS is a biphasic form of both sarcomatous spindle cell components and epithelial components, the latter being glandular, solid nests, or squamous differentiation [3]. SS can be subtyped into monophasic, where only the spindle cell component is appreciated, or the calcifying subtype [3]. And others were considered poorly differentiated (or round cell) subtype [5]. Micro-calcifications are described in up to 60% of the cases [4]. Calcification can be an important radiological clue, too [3]. 

Notably, TLE1, BCL2, and vimentin positivity in both spindle and epithelial cells is a key diagnostic feature [5], with cytokeratin expression observed in both components in approximately 90% of cases [3]. However, FISH and RT-PCR confirmation of the SS18 rearrangement remains the most definitive diagnostic test [6]. 

Our case is distinctive due to its incidental perioperative discovery in an elderly patient, histologically biphasic morphology with epithelial differentiation and neuroendocrine-like features, and the absence of clinical or radiologic evidence of metastasis at diagnosis. This contrasts with previous reports, where younger patients typically presented with symptomatic, ulcerated, or rapidly recurring tumors, often with lymph node involvement. While SS of the floor of the mouth are known for their heterogeneous clinical and histopathologic presentations, our case further expands this spectrum by highlighting an older patient demographic, prominent epithelial pleomorphism, and neuroendocrine-like immunophenotypic elements. We compared our case to others arising from the floor of the mouth or adjacent mucosa (Table 1). 

**Table 1:** Summary of the clinical and histological features of previously reported cases of floor of mouth/buccal synovial sarcoma

Case number	Reference	Oral Site	Age /Gender	Size of the Lesion (cm)	Clinical Presentation	Growth Pattern/Cell Morphology	Molecular Studies	Treatment Approach	Follow-up and Outcome
1	Mahesh et al. (2013) [5]	Buccal mucosa	24/M	12.0	Mass with compressive symptoms	Poorly differentiated small round cell tumor	Not specified	Patient discontinued treatment	Death after one year
2	Desai et al. (2023) [7]	Alveolar mucosa	35/F	5.0	Ulcerated mass	Monophasic	Not specified	Surgery with adjuvant radiotherapy	Metastasis after three months to the lungs and death within one month
3	Dholaria et al. (2017) [1]	Alveolar mucosa	33/M	4.0	Ulcer	Undifferentiated	Not specified	Surgery with neck dissection, adjuvant radiotherapy	Not specified
4	Wang et.al. (2020) [6]	Floor of the mouth	45/M	5.9	Ulcerating mass	Monophasic	Not specified	Surgery	No recurrence at the 46-month follow-up
5	de Araújo et al. (1989) [8]	Buccal mucosa	36/M	2.0	Painless mass	Biphasic	Not specified	Surgery	No recurrence at the seven-month follow-up
6	Shabnum et. al. (2003) [9]	Floor of the mouth	49/M	4.5	Painful exophytic lesion	Biphasic	Not specified	Surgery	The patient was lost to follow-up.
7	Jayasooriya et al. (2016) [10]	Alveolar mucosa	53/F	3.5	Exophytic ulcerating mass	Monophasic	Not specified	Not specified	Not specified
8	Anand et al. (2008) [11]	Floor of the mouth	20/M	Not specified	Painless swelling	Biphasic	Not specified	Chemotherapy and radiotherapy	No recurrence at the 10-month follow-up
9	Shmookler et al. (1982) [12]	Buccal mucosa	35/F	2.5	Gradually enlarging mass	Biphasic	Not specified	Surgery	No recurrence at the 1.3-year follow-up

One such case [7] involved a 45-year-old male patient with a 5.9 cm right floor-of-mouth mass initially misdiagnosed as fibroadenoma, later showing rapid recurrence and lymph node metastases. Immunohistochemistry revealed positivity for TLE-1, BCL2, and CD99, but negativity for p63, CK, and S100, findings that largely overlap with our case. However, unlike the progressive course observed in that case, our patient had no evidence of nodal involvement or metastatic disease at presentation. Similarly, in Case 3 [9], a 49-year-old male patient with a 4.5 cm painful ulcerated lesion in the floor of the mouth demonstrated a biphasic histologic pattern with spindle cells interspersed with gland-like structures. Immunohistochemistry showed CK7, EMA, BCL2, and CD99 positivity, paralleling some features of our case, though our tumor was biphasic with predominantly epithelial differentiation and neuroendocrine morphology. 

A notable histologic distinction in our case was the presence of highly pleomorphic epithelial nests with neuroendocrine-like immunohistochemical features (focal synaptophysin and CD56 positivity), an uncommon finding in prior reports. For instance, in Case 9 [7], a 35-year-old female with a locally advanced mass in the lower anterior alveolus exhibited a monophasic SS with a hemangiopericytomatous pattern but lacked the biphasic growth and neuroendocrine-like features observed in our case. 

The recommended choice of up-to-date management is a wide surgical excision after adjuvant radiation with or without chemotherapy. In Case 7 [1], the patient underwent left hemimandibulectomy with neck dissection and fibula free flap reconstruction, followed by adjuvant radiation therapy, resulting in a disease-free outcome. Similarly, in Case 4 [10], the patient received chemotherapy and radiotherapy, leading to a significant reduction in tumor size and no evidence of recurrence at follow-up. However, in our case, the patient was deemed unfit for surgery and chemotherapy due to comorbidities and was managed with palliative radiotherapy, which resulted in a disease-free status within nine months during palliative care stay. This outcome remains to question if radiotherapy can be an effective alternative in patients who are not candidates for surgery or chemotherapy, as long-term prognosis of SS in the oral cavity is guarded, as highlighted in Case 9 [7], where the patient developed metastatic disease and succumbed to the illness despite aggressive treatment. 

Unlike most cases managed with surgery and adjuvant therapy, this patient’s initial care focused on an incidental staghorn calculus with cortical scarring, delaying attention to the oral lesion. This case illustrates how oral SS can go unnoticed, especially when overshadowed by unrelated findings like renal calculi. The incidental nature of the tumor, the diagnostic challenges, and the decision to manage it with palliative radiotherapy highlight the value of a multidisciplinary approach in recognizing and treating SS [12]. Further studies are needed to better understand the optimal management strategies for this rare and aggressive tumor. 

## Conclusions

This report adds to the limited literature on oral SS by documenting a rare, incidentally discovered biphasic tumor with neuroendocrine-like features in an elderly patient. To our knowledge, this is the first reported case of oral SS in Saudi Arabia or the surrounding region. It emphasizes the importance of comprehensive head and neck examinations during unrelated medical evaluations and advocates for multidisciplinary collaboration in managing complex and aggressive neoplasms. 
